# Prevention of oral mucositis with cryotherapy in children undergoing hematopoietic stem cell transplantations—a feasibility study and randomized controlled trial

**DOI:** 10.1007/s00520-019-05258-2

**Published:** 2020-01-28

**Authors:** Tove Kamsvåg, Anncarin Svanberg, Karin Garming Legert, Johan Arvidson, Louise von Essen, Karin Mellgren, Jacek Toporski, Jacek Winiarski, Gustaf Ljungman

**Affiliations:** 1 Department of Women’s and Children’s Health, Pediatric Oncology, Uppsala University, Uppsala, Sweden; 2 Department of Medical Sciences, Uppsala University, Uppsala, Sweden; 3 Department of Dental Medicine, Karolinska Institutet, Stockholm, Sweden; 4 Department of Women’s and Children’s Health, Clinical Psychology in Healthcare, Uppsala University, Uppsala, Sweden; 5 Department of Pediatric Oncology, Institute of Clinical Sciences, University of Gothenburg, Gothenburg, Sweden; 6 Department of Clinical Sciences, Pediatric Oncology and Hematology, University of Lund, Lund, Sweden; 7 Department of Pediatrics, Astrid Lindgren’s Children’s Hospital, Karolinska University Hospital, Huddinge, Stockholm, Sweden; 8 Department of Clintec, Karolinska Institutet, Stockholm, Sweden

**Keywords:** Oral mucositis, Children, Hematopoietic stem cell transplantation, Oral cryotherapy, Feasibility

## Abstract

**Purpose:**

To evaluate the feasibility of oral cryotherapy (OC) in children and to investigate if OC reduces the incidence of severe oral mucositis (OM), oral pain, and opioid use in children undergoing hematopoietic stem cell transplantation (HSCT)*.*

**Methods:**

Fifty-three children, 4–17 years old, scheduled for HSCT in Sweden were included and randomized to OC or control using a computer-generated list. OC instructions were to cool the mouth with ice for as long as possible during chemotherapy infusions with an intended time of ≥ 30 min. Feasibility criteria in the OC group were as follows: (1) compliance ≥ 70%; (2) considerable discomfort during OC < 20%; (3) no serious adverse events; and (4) ice administered to all children. Grade of OM and oral pain was recorded daily using the WHO-Oral Toxicity Scale (WHO-OTS), Children’s International Oral Mucositis Evaluation Scale, and Numerical Rating Scale. Use of opioids was collected from the medical records.

**Results:**

Forty-nine children (mean age 10.5 years) were included in analysis (OC = 26, control = 23). The feasibility criteria were not met. Compliance was poor, especially for the younger children, and only 15 children (58%) used OC as instructed. Severe OM (WHO-OTS ≥ 3) was recorded in 26 children (OC = 15, control = 11). OC did not reduce the incidence of severe OM, oral pain, or opioid use.

**Conclusion:**

The feasibility criteria were not met, and the RCT could not show that OC reduces the incidence of severe OM, oral pain, or opioid use in pediatric patients treated with a variety of conditioning regimens for HSCT.

**Trial registration:**

ClinicalTrials.gov id: NCT01789658

**Electronic supplementary material:**

The online version of this article (10.1007/s00520-019-05258-2) contains supplementary material, which is available to authorized users.

## Introduction

Oral mucositis (OM) is a common adverse effect of antineoplastic treatment [1, 2]. The condition often causes pain and difficulties in basal functions, such as talking and swallowing, which in turn affect drinking and eating. OM is reported to be one of the most painful and debilitating side effects of cancer treatment in pediatric patients [3–5]. The incidence of OM in children ranges between 52 and 81% depending on the type of antineoplastic treatment given [6, 7]. The majority of patients undergoing hematopoietic stem cell transplantation (HSCT) develop some degree of OM [2]. From a healthcare perspective, OM delays treatment, which reduces its intensity and increases the incidence of infections, total parental nutrition use, drug consumption, and hospitalization [1]. As well as causing an increased morbidity and suffering, OM also increases healthcare costs and mortality [8].

Preventive interventions and therapeutic treatments for OM in adults have been evaluated in Cochrane reviews [9, 10] and by the Mucositis Study Group of the Multinational Association of Supportive Care in Cancer and the International Society of Oral Oncology [11]. The few guidelines for the treatment of OM in children recommend standard oral care protocols and conclude that further research is needed to evaluate other interventions infrequently used in children [12, 13].

Oral cryotherapy, that is the cooling of the mouth during chemotherapy infusions, was first shown to prevent oral mucositis in adults treated with 5-FU in 1991 [14]. The mechanism behind cryotherapy is only somewhat understood. The theory is that oral cryotherapy (OC) promotes vasoconstriction resulting in reduced drug delivery and hence less tissue toxicity [14, 15]. In adults, there is evidence that OC may reduce the incidence and severity of OM in patients treated with HSCT, especially when melphalan is given as the conditioning agent [16–18]. To our knowledge, no studies have evaluated the effect of OC in children. Sung et al. give a weak recommendation for the use of OC in children based on the potential benefits of the treatment and low risk of harm [13]. For some patients, the treatment can be difficult considering the unpleasantness of the cold therapy and there is as yet little knowledge of children’s ability to conduct OC.

The primary objectives of this study are to evaluate the feasibility of OC in children and to investigate if OC decreases the grade of OM, oral pain, and use of opioids in children undergoing HSCT. Secondary objectives are to explore if OC has a positive effect on the child’s nutritional status, decreases the risk of infections, and shortens hospitalization for children undergoing HSCT.

## Materials and methods

### Design

The study is a feasibility and randomized controlled clinical trial. Due to the nature of the intervention, the participants and nurses were not blinded. However, the outcome assessors and data analysts were kept blinded to the allocation. The Regional Ethical Review Board in Uppsala, Sweden, approved the study (Dnr 2012/126) which was registered at ClinicalTrials.gov (ClinicalTrials.gov id: NCT01789658).

### Participants

In Sweden, with a population of 10 million inhabitants, approximately 50 children undergo HSCT every year at four departments. Swedish-speaking children between 4 and 17 years admitted for autologous or allogeneic HSCT were eligible to participate in the study. The transplant coordinator at each department identified eligible children. Children were included consecutively between September 2012 and June 2016.

### Oral cryotherapy

The children in the OC group were instructed to cool their mouths by sucking on ice chips and ice popsicles or rinsing their mouths with ice cold water, depending on their preference, during chemotherapy infusions given as conditioning treatment. A nurse or an assistant nurse made the ice on the ward by freezing water with/without flavoring. The ice was then crushed into ice chips and served in a clean ice bucket when the chemotherapy infusion started. The child was instructed to cool his/her mouth for as long as possible during the chemotherapy infusions and the nurse encouraged the child to use OC for at least 30 min. Children with > 12-h infusions were instructed to cool their mouth four times a day beginning when the infusion started. After each session, a nurse documented the time the child had cooled his/her mouth and after each day of chemotherapy/OC, the child together with his/her parents answered a questionnaire evaluating the therapy (see Online Resource 1).

### Routine oral care

All the children received standard oral care including examination of the oral cavity and if necessary dental treatment prior to start of chemotherapy and daily oral examinations by a nurse during hospitalization. The child and parents were instructed, if possible, to gently brush the child’s teeth twice a day using a soft toothbrush and mild toothpaste, to wash the mouth with saline after meals, and to avoid toothpicks and dental floss. Discomfort from the mouth was treated according to local clinical guidelines. Hospital dentists were consulted if needed during the hospitalization.

### Pain relief

The children received pain relief according to local clinical guidelines. In general, pain relief is based on paracetamol with the addition of oral or intravenous opioids when required either as intermittent doses or continuous infusion. Local treatment, for example lidocaine viscous solution, benzydamide, and hyaluronic acid, was used dependent on the child’s preferences.

### Parenteral nutrition

Most children have a nasogastric tube or percutaneous endoscopic gastrostomy for nutritional support during the treatment. Calorie intake was calculated daily and TPN was given if needed.

### Outcome measurements

The feasibility criteria for success were as follows: (1) 70% should comply ≥ 70% of the days, (2) less than 20% should report considerable discomfort during OC, (3) no serious adverse events should occur, and (4) ice/ice pop should be administered to all children randomized to OC. Compliance was defined as the percentage of days with chemotherapy that the child used OC for a minimum of 30 min based on previous studies on adults and the average time of chemotherapy infusion the children received.

Mucositis was measured using the WHO-Oral Toxicity Scale (WHO-OTS), scoring from 0 to 4 [19]. Severe OM was defined as a WHO-OTS score ≥ 3. All nurses had received special training in the assessment and grading of mucositis by the study coordinator and it was calibrated using eight different cases. The child recorded symptoms of mucositis by using the Children’s International Mucositis Evaluation Scale (ChIMES) [20, 21]. The scale consists of seven items: (1) amount of mouth and/or throat pain, (2) effect of mouth and/or throat pain on swallowing, (3) effect of mouth and/or throat pain on eating, (4) effect of mouth and/or throat pain on drinking, (5a) use of pain medication, (5b) use of pain medication due to mouth and/or throat pain, and (6) presence of ulcers. ChIMES questions 1–4 are graded 0–5, questions 5a and 5b are graded 1 if the child has received pain medication, and question 6 is graded 1 if ulcers are present. The ChIMES total score is calculated by adding the scores for each question (missing questions and the answer “I don’t know” are scored 0), giving a maximum score of 23. The scale has shown good reliability, content validity, and acceptability for children undergoing HSCT [22]. The original English scale was translated into Swedish using a forward-backward procedure [23]. Since mouth and throat pain are not separated in ChIMES, children 4–6 years old recorded mouth pain separately with the Faces Pain Scale Revised (FPS-R) [24, 25] and children ≥ 7 years old by using the Numerical Rating Scale (NRS) [25, 26]. Both scales, hereafter referred to as NRS, are scored 0–10. Parents reported proxy ratings using the parent version of ChIMES and NRS for mouth pain.

The use of opioids was collected from the participants’ medical record. The total dose of opioids was calculated by converting both p.o. morphine and other opioids, such as oxycodone, into comparable i.v. morphine doses (mg). The primary outcomes were the incidence of severe OM, the area under the curve (AUC) for WHO-OTS, ChIMES total and NRS scores within 24 days post transplantation, days with opioids, and total dose of opioids per kilo.

Secondary outcomes were collected from the participants’ medical records. Days until engraftment (neutrophil count ≥ 0.5 × 10^9^/L and platelet count ≥ 0.5 × 10^9^ for two consecutive days) and days until discharge were calculated from the day for stem cell transfusion (day 0). Fever was defined as a single axillary or oral temperature measurement of > 38.0 °C. Infection rate was assessed based on the presence of neutropenic fever, level of C-reactive protein (CRP), and use of i.v antibiotics. Nutritional status was assessed based on weight change and administration of TPN and s-albumin (reference values 37–48 g/L).

### Procedure

Children and parents/guardians were informed and invited to participate in the study in conjunction with admission to the HSCT department by the transplant coordinator or research nurse. After consent and assent had been obtained, randomization to OC or control was done by the telephone, using a computer-generated list, by the study controller not involved in the care of the patient.

Baseline assessment was performed on the day of hospital admission and daily assessments started on the day for stem cell transfusion (day 0). Mouth pain and mucositis were reported daily by the children, parents, and nurses using the above-mentioned instruments from day 0 until engraftment. Data on pain, infectious, and nutritional status were collected during the same period from the child’s medical chart.

### Statistical analysis

Previous studies have suggested that a change of 13–18 mm for pain on a visual analogue scale (VAS) would indicate a clinically relevant difference [27, 28]. We set 15 mm as a reasonable effect size. Twenty-five children were needed in each group to demonstrate a difference between cryotherapy and control with a power of 0.80 and an alpha value of 0.05, assuming a standard deviation of 20 mm as found in earlier studies [29, 30].

Data were analyzed and reported according to the CONSORT guidelines [31]. Chi-square, Mann-Whitney *U* test, and Spearman’s rank order correlation were used for feasibility analyses in the oral cryotherapy group. Two children did not receive allocated intervention and two children were lost to follow-up and were removed from the final intention to treat (ITT) analysis [32]. Multiple imputation methods were used to impute missing outcomes for WHO-OTS, ChIMES, and NRS. One hundred imputed data sets were created and pooled for analysis using the R-package mice. Analysis was carried out separately for each 100 imputed data sets and pooled using Rubin’s rules [33]. Comparative analyses were made with the unimputed data and showed similar results. To describe differences between the groups, the independent 2-sample *t* test was used for numerical variables and chi-square and logistic regression for categorical data. The statistical analyses were performed using SPSS 23.0 and R 3.4.0. A *p* value of < 0.05 was regarded as a statistically significant difference.

## Results

### Participants and background data

**Fig. 1:**
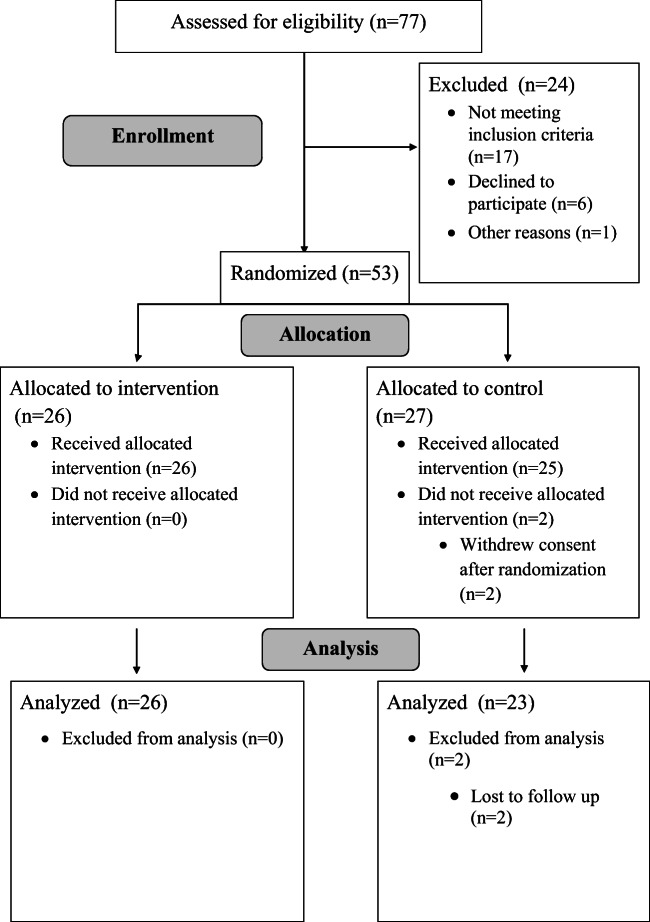
Flowchart for enrollment, allocation, and analysis

Seventy-seven children were consecutively assessed for eligibility. Fifty-three children were included in the study. Two children declined participation after randomization to the control group and used oral cryotherapy at will, one child was admitted to the intensive care unit, and one child was cared for at home within the first days after transplant resulting in lack of data and was therefore removed from the analyses. The final sample consisted of 49 children (mean age 10.5 ± 4.3 years). Twenty-six children were randomized to the OC group (mean age 11.3 ± 3.8 years) and 23 to the control group (mean age 9.5 ± 4.6 years) (see Fig. 1). Background data is presented in Table 1.

**Table 1:** Gender, age, treatment, and diagnosis of children presented as numbers (%)

	Cryotherapy *n* = 26 (%)	Control *n* = 23 (%)	Total *n* = 49 (%)
Gender			
Male	15 (57.7)	11 (47.8)	26 (53.1)
Female	11 (42.3)	12 (52.2)	23 (46.9)
Age group			
< 7 years	4 (15.4)	7 (30.4)	11 (22.4)
≥ 7 years	22 (84.6)	16 (69.6)	38 (77.6)
HSCT			
Allogeneic RD	10 (38.5)	6 (26.1)	16 (32.6)
Allogeneic URD	11 (42.3)	13 (56.5)	24 (49.0)
Autologous	5 (19.2)	4 (17.4)	9 (18.4)
Diagnosis			
ALL	10 (38.5)	5 (21.7)	15 (30.6)
AML	2 (7.7)	2 (8.7)	4 (8.2)
CML	0 (0)	2 (8.7)	2 (4.1)
Lymphoma	3 (11.5)	3 (13.0)	6 (12.2)
Neuroblastoma	1 (3.8)	2 (8.7)	3 (6.1)
Ewing sarcoma	1 (3.8)	0 (0)	1 (2.0)
MDS	2 (7.7)	2 (8.7)	4 (8.2)
HLH	0 (0)	1 (4.3)	1 (2.0)
SAA	1 (3.8)	0 (0)	1 (2.0)
Fanconi anemia	2 (7.7)	2 (8.7)	4 (8.2)
Thalassemia	2 (7.7)	2 (8.7)	4 (8.2)
Sickle cell disease	1 (3.8)	1 (4.3)	2 (4.1)
MS	1 (3.8)	1 (4.3)	2 (4.1)

*RD* related donor, *URD* unrelated donor, *ALL* acute lymphoblastic leukemia, *AML* acute myeloid leukemia, *CML* chronic myeloid leukemia, *MDS* myelodysplastic syndrome, *HLH* hemophagocytic lymphohistiocytosis, *SAA* severe aplastic anemia, *MS* multiple sclerosis

There were no differences between the OC and control groups regarding gender, age, and diagnoses. Conditioning regimens used (Online Resource 2) showed no differences between the two groups. All the children scored 0 on the WHO-OTS at admission.

### Feasibility of oral cryotherapy

Five children allocated to OC received chemotherapy for 1 day, five for 2–4 days, and sixteen for 5 days or more providing a total of 130 days of possible cryotherapy sessions in the group. (See Table 2 for details for each child in the OC group.) Cryotherapy was used ≥ 30 min in 74 sessions. In the other sessions, OC was used for less than 30 min in 6 sessions (24–10 min) and in 50 sessions, OC was not used at all. To cool the mouth, a combination of ice popsicles and ice chips was used in 24 sessions, in 14 sessions only ice chips were used, and in 8 sessions only ice popsicles were used. Ice water was used in one session (missing observations 27). There was no difference in compliance depending on how OC was administered.

**Table 2:** Age, days receiving chemotherapy (CT), and days with/without oral cryotherapy (OC) ≥ 30 min

Child	Age	Days with CT	Days with OC	Days without OC
1	14	1	1	0
2	12	5	3	2
3	16	1	1	0
4	7	4	3	1
5	12	7	0	7
6	13	6	1	5
7	4	5	2	3
8	14	9	7	2
9	16	6	6	0
10	5	3	0	3
11	14	5	5	0
12	16	6	3	3
13	6	1	0	1
14	10	4	4	0
15	7	1	1	0
16	10	5	4	1
17	14	1	1	0
18	10	8	0	8
19	11	5	2	3
20	14	3	3	0
21	13	6	1	5
22	4	8	0	8
23	12	1	1	0
24	10	8	6	2
25	16	13	13	0
26	14	6	6	0

Compliance with OC ranged between 0 and 100% with a median of 77% (SD = 40, range 0–100). In total, 15 children (58%) met the feasibility criteria of ≥ 70% compliance. Children who used cryotherapy did so for 10–255 min a day (*m* = 98 min, Md = 60 min). All the children who only received chemotherapy for 1 day had a compliance of 100%. No difference in compliance was found based on gender, diagnosis, type of HSCT, conditioning regimen used, or if the child received chemotherapy for 2–4 days or ≥ 5 days. There was a trend indicating that children who were treated with busulfan as part of their conditioning regimen had lower compliance than children not treated with busulfan (*m* = 50%, Md = 55% versus *m* = 74%, Md = 100%, *p* = 0.08). Compliance correlated with age (*r* = 0.56 *p* = 0.003) with better compliance in older children. Children younger than 7 years of age were less compliant with OC than older children (Md 0%, SD 10 versus Md 90%, SD 35, *p* = 0.007).

**Fig. 2:**
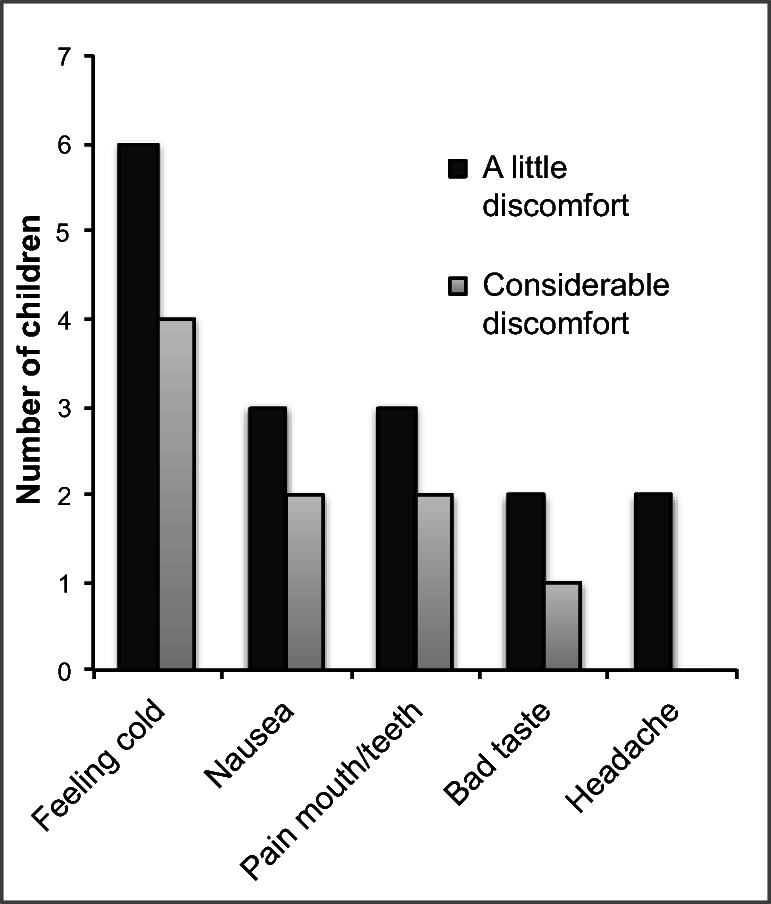
Discomfort and degree of discomfort reported after oral cryotherapy. The same child could report more than one type of discomfort

The most common reasons for not using cryotherapy were the following: discomfort (*n* = 11), nausea (*n* = 9), being asleep (*n* = 8), forgetting to cool the mouth (*n* = 6), others (*n* = 4), and missing (*n* = 16). Thus, ice was not administered to the children during all the OC sessions. A little discomfort was reported by four children and considerable discomfort by seven children after one or more sessions of OC (see Fig. 2). No severe adverse events were reported.

### Grade of mucositis and ChIMES score

Oral mucositis was recorded in 39 (80%) children and severe OM in 26 children (15 and 11 children in the OC group and control group respectively). There was no difference in incidence of severe OM between children undergoing allogeneic (15 of 40 children) and autologous HSCT (5 of 9 children). There were no differences in the incidence of severe mucositis among children with TBI (7 of 15 children), melphalan (6 of 10 children), or busulfan (11 of 20 children) as part of the conditioning regimen compared with the other children. Children who received fludarabine as part of the conditioning regimen showed a lower grade of severe OM (4 of 16 children, *p* = 0.034). The children with a malignancy showed higher incidence of severe OM than the children with a benign disease (53% versus 18%, *p* = 0.03).

**Fig. 3:**
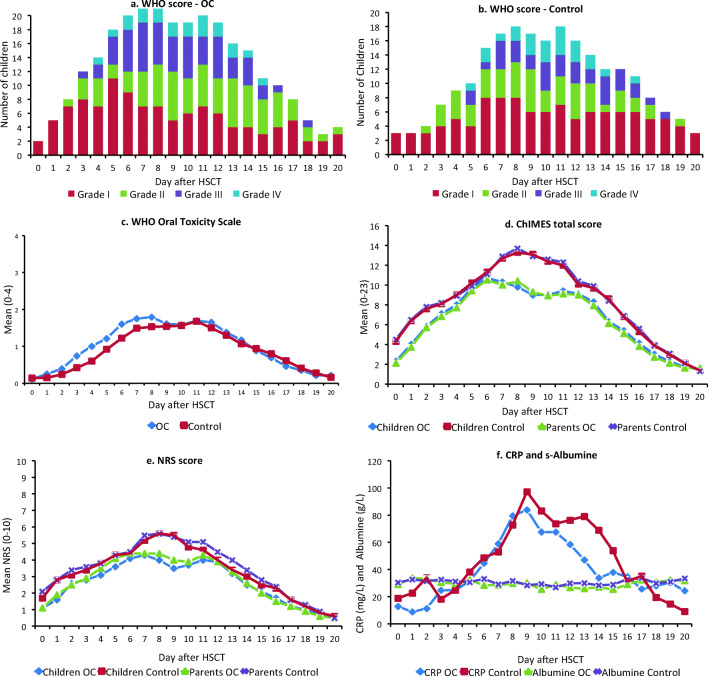
Daily measurements for day 0–20 after HSCT for OC and control group respectively. **a** Grade of oral mucositis according to WHO-OTS in the OC group (*n*). **b** Grade of OM according to WHO-OTS in the control group. **c** Mean WHO-OTS score. **d** Children’s and parents’ proxy, mean ChIMES total score. **e** Children’s and parents’ proxy, mean NRS-score for oral pain. **f** CRP and s-albumin values. OC oral cryotherapy, WHO-OTS WHO Oral Toxicity Scale, ChIMES Children’s International Mucositis Evaluation Scale, NRS Numerical Rating Scale, CRP C-reactive protein

There were no differences between children in the OC group and control group regarding grade of mucositis, ChIMES, or NRS scores (see Fig. 3a–e and Table 3, section a).

**Table 3:** Primary and secondary outcomes: (a) incidence of severe oral mucositis and AUC for grade of OM, children’s and parents’ proxy ChIMES total and NRS for oral pain score; (b) opioid use during the neutropenic phase; (c) nutritional and infectious parameters during the neutropenic phase. *OM* oral mucositis, *WHO-OTS WHO*-Oral Toxicity Scale, *ChIMES* Children’s International Mucositis Evaluation Scale, *NRS* Numerical Rating Scale, *TPN* total parenteral nutrition

	Oral cryotherapy (*n* = 26)	Control (*n* = 23)	*p* value
a.			
Severe OM (grades 3–4), *n* (%)	15 (57.7)	11 (47.8)	0.43
Days with severe OM, mean (SD)	3.2 (3.7)	2.7 (4.2)	0.68
AUC^a^ for WHO-OTS score (SD)	21.2 (14.8)	18.9 (15.6)	0.61
AUC^a^ for ChIMES total children (SD)	140.0 (128.5)	174.9 (108.4)	0.31
AUC^a^ for ChIMES total parents (SD)	136.9 (105.8)	176.2 (131.6)	0.26
AUC^a^ for oral pain (NRS) children (SD)	56.2	70.0	0.38
AUC^a^ for oral pain (NRS) parents (SD)	59.1	74.1	0.33
b.			
Days with opioids (SD)	8.4 (7.2)	8.3 (7.2)	0.34
Total dose of opioids^b^, mg (SD)	168.1 (232.5)	170.2 (271.1)	0.98
Total dose of opioids^b^, mg/kg (SD)	4.5 (6.7)	5.0 (7.1)	0.76
c.			
Weight change, kg (SD)	− 0.3 (2.4)	+ 0.2 (1.4)	0.38
Days with TPN (SD)	9.0 (8.9)	6.6 (7.0)	0.35
Days with fever (SD)	5.8 (5.8)	3.2 (3.2)	0.08
Days on antibiotics (SD)	12.7 (8.0)	12.1 (5.6)	0.75

^a^Mean values day 0–24 after HSCT

^b^IV morphine equivalents

### Use of opioids

Thirty-eight children (21 in the OC group and 17 in the control group) were treated with opioids (see Table 3, section b). Children with severe OM were treated with significantly more opioids (mean 13 days, 8.8 mg/kg versus 5 days, 1.9 mg/kg, *p* < 0.001 for both) than the children with no/milder OM.

### Days until engraftment, nutritional status, and infections

On average, engraftment was recorded at 19.7 ± 4.2 days and the children spent 30.0 ± 17.6 days at the hospital. There were no differences between the children in the OC and the control group. Nutritional and infectious parameters are reported in Table 3 sections b and c and Fig. 3e. Children with severe OM used TPN for 11 days in contrast to the other children who used it for 6 days (*p* = 0.048).

## Discussion

In adults, OC has been shown to be useful for patients undergoing HSCT; however, conclusive studies in children are missing. The accessibility, low cost, and lack of serious side effects make the intervention appealing for pediatric patients. In this study, we found that the feasibility of OC among the children was lower than expected, especially for younger children and only 15 children (58%) met the feasibility criterion. We could not find that OC reduced the grade of OM, oral pain, or use of opioids in children undergoing HSCT in this study.

There are few studies on feasibility and compliance with OC. Svanberg et al. [18] used OC in adults undergoing HSCT and reported that 58–75% of the patients had used OC according to the protocol dependent on conditioning regimes. Sorensen et al. [34] used OC for 45 min in 67 adult patients and reported that 87% of the patients were able to use OC according to the protocol. Sato et al. [35] used OC in 13 children but compliance is not reported. However, the study reports that some younger children were not able to perform OC due to being asleep.

We found that the children had more difficulties in complying with the intervention than expected. Discomfort was the most common reason for not using OC but nausea and being asleep also prevented the children from using OC as instructed. However, not all the children reported discomfort from the intervention and some managed to use OC for up to 4 h, indicating that the acceptability of the treatment is individual. All the children only receiving chemotherapy for 1 day had a compliance of 100%. For the other children, no difference in compliance was seen based on the number of days with chemotherapy. In general, the children either accepted the treatment and were able to use OC for >30 min or only tried the interventions for a few minutes or not at all. The children treated with busulfan showed a trend of lower compliance than those children not treated with busulfan. Busulfan is often administered four times a day in 2-h infusions for several days, which might explain why children receiving busulfan as part of their conditioning regimen showed a trend of lower compliance. The intervention was particularly troublesome for younger children, but there were only four children under 7 years of age included in the study, which makes it difficult to draw firm conclusions. We also found that ice had not been administered consistently to all the children. This could be since the children on previous days had refused to use OC, but it also reflects the clinical reality in a busy ward.

The exact time needed for oral cooling to have an effect on OM is not known and the preset criteria of 30 min and 70% of the chemotherapy sessions were operationally defined after discussions with clinicians and specialists in the area, taking into account scientific evidence and the possible difficulties of getting children to accept the intervention. We chose to include children from 4 years of age given that most 4-year-olds are able to understand and follow instructions from parents and nurses. The results indicate that the age limit was too low and that a more suitable age limit might have been 7 years of age.

The study provides a picture of the course of mucositis during HSCT with a peak on days 8–9 for WHO-OTS, ChIMES total, and NRS scores. This is consistent with the course of neutropenia and coincides with the CRP peak, affirming previous studies on OM during HSCT [36]. We found relatively high scores for oral pain despite high doses of opioids for pain relief being used. The children who developed severe OM were treated with significantly more opioids and TPN than those with no or milder OM, confirming the suffering and clinical challenges of OC in children. We found that children with a malignant disease had a higher incidence of severe OM than the children with a benign disease. This is likely an effect of the different conditioning regimens used. We could see that fludarabine-based regimens showed lower incidence of severe OM, consistent with previous studies [37].

Given the high incidence of OM in children undergoing HSCT independently of conditioning regimens used, we decided to include all children undergoing HSCT to acquire as large a sample as possible. A consequence of this is that the sample is heterogeneous in terms of diagnoses and conditioning regimens, which could affect risk and grade of OM. There are studies evaluating OC on a group level in adults undergoing HSCT that have shown beneficial effects of the intervention [18]. However, most evidence in adults supports the use of OC primarily with chemotherapy regimens with short plasma half-life such as melphalan and 5-FU [9, 11]. In this study, OC was mostly used in chemotherapy regimens that differed from those in evidence-based guidelines recommending OC for the prevention of OM. In the studies underpinning these guidelines, OC is used for 30–60 min, which usually does not cause compliance problems. Compliance with OC administered multiple times a day and for several days seems much more challenging, particularly in young children. Since the number of children who received agents with short plasma half-life in our study was low (10 children received melphalan), it is difficult to draw firm conclusions about the effect of OC in these specific treatments. As in all multicenter trials, there is a risk of variations in compliance to the study protocol. The study coordinator made frequent visits to the different centers to minimize this risk. The final sample consisted of 49 children, which is close to the preset goal based on the power analysis. However, with a larger sample, we could have performed subgroup analyses that might have provided interesting results.

Many of the children were not able to use OC as instructed and the effect size would have been overestimated if only children who complied with OC had been analyzed. Especially in children, it is important not only to assess the results of an intervention but also the feasibility and safety aspects of the treatment. For some children, data regarding grade of mucositis and oral pain was missing for one or more days. This is a common problem in clinical trials. We chose to use multiple imputations to handle missing data based on recommendations in the literature to maintain power [38]. As a control, analyses were also performed without imputations and the results showed good consistency.

We collected data from children, parents (ChIMES and NRS), and nurses (WHO-OTS) to get as comprehensive information as possible and used outcomes according to suggestions in PedIMMPACT [25]. The nurses evaluating OM were trained and validated using the WHO-OTS; nevertheless, examinations of children’s mouths can be difficult. The scale takes into account the patients’ ability to eat. However, eating is often troublesome for many other reasons over and above mucositis, for example nausea, other pain, and fever during the treatment. The lack of blinding of the intervention might have influenced the nurses’, children’s, and parents’ evaluations. Maybe it would have been better to let a “blinded” nurse or dentist evaluate OM but for practical reasons, this was considered challenging to perform in the study and to be of limited value.

The intervention is difficult to blind but the fact that the control group received no treatment apart from standard care might have added a placebo effect for the child and parent in the OC group. Two children dropped out of the study shortly after randomization to the control group because they were eager to try OC to possibly reduce the risk of severe OM. This shows an expectation that the intervention, which had already been implemented as standard care for adults undergoing HSCT in some transplant centers in Sweden, might help them.

## Conclusions

The feasibility criteria were not met. The compliance with OC was lower than expected, and especially the younger children had difficulties accepting the intervention. We could not see any indication that OC reduced the incidence of OM in children undergoing HSCT in this study. However, the heterogeneity of diagnoses, conditioning regimens, age, and the low compliance with OC makes it difficult to draw any firm conclusions. For future studies of OC in children, it would be preferable to have a more homogenous sample. This would, by necessity, require multinational collaboration.

## Electronic supplementary material

ESM 1 (PDF 79 kb)

ESM 2 (PDF 63 kb)

## References

[1] Sonis ST, Elting LS, Keefe D, Peterson DE, Schubert M, Hauer-Jensen M, Bekele BN, Raber-Durlacher J, Donnelly JP, Rubenstein EB (2004). Perspectives on cancer therapy-induced mucosal injury: pathogenesis, measurement, epidemiology, and consequences for patients. Cancer.

[2] Vagliano L, Feraut C, Gobetto G, Trunfio A, Errico A, Campani V, Costazza G, Mega A, Matozzo V, Berni M, Alberani F, Banfi MM, Martinelli L, Munaron S, Orlando L, Lubiato L, Leanza S, Guerrato R, Rossetti A, Messina M, Barzetti L, Satta G, Dimonte V (2011). Incidence and severity of oral mucositis in patients undergoing haematopoietic SCT-results of a multicentre study. Bone Marrow Transplant.

[3] Cheng KK, Lee V, Li CH, Yuen HL, Epstein JB (2012). Oral mucositis in pediatric and adolescent patients undergoing chemotherapy: the impact of symptoms on quality of life. Support Care Cancer.

[4] Kamsvag-Magnusson T, Thorsell-Cederberg J, Svanberg A, von Essen L, Arvidson J, Mellgren K, Toporski J, Ljungman G (2014). Parents and children’s perceptions of distress related to oral mucositis during haematopoietic stem cell transplantation. Acta Paediatr.

[5] Ljungman G, Gordh T, Sorensen S, Kreuger A (2000). Pain variations during cancer treatment in children: a descriptive survey. Pediatr Hematol Oncol.

[6] Fadda G, Campus G, Luglie P (2006). Risk factors for oral mucositis in paediatric oncology patients receiving alkylant chemotherapy. BMC Oral Health.

[7] Cheng KK, Lee V, Li CH, Goggins W, Thompson DR, Yuen HL, Epstein JB (2011). Incidence and risk factors of oral mucositis in paediatric and adolescent patients undergoing chemotherapy. Oral Oncol.

[8] Sonis ST, Oster G, Fuchs H, Bellm L, Bradford WZ, Edelsberg J, Hayden V, Eilers J, Epstein JB, LeVeque F, Miller C, Peterson DE, Schubert MM, Spijkervet FK, Horowitz M (2001). Oral mucositis and the clinical and economical outcomes of hematopoietic stem-cell transplantation. J Clin Oncol.

[9] Worthington HV, Clarkson JE, Bryan G, Furness S, Glenny AM, Littlewood A, McCabe MG, Meyer S, Khalid T (2011). Interventions for preventing oral mucositis for patients with cancer receiving treatment. Cochrane Database Syst Rev.

[10] Clarkson JE, Worthington HV, Furness S, McCabe M, Khalid T, Meyer S (2010). Interventions for treating oral mucositis for patients with cancer receiving treatment. Cochrane Database System Rev.

[11] Lalla RV, Bowen J, Barasch A, Elting L, Epstein J, Keefe DM, McGuire DB, Migliorati C, Nicolatou-Galitis O, Peterson DE, Raber-Durlacher JE, Sonis ST, Elad S (2014). MASCC/ISOO clinical practice guidelines for the management of mucositis secondary to cancer therapy. Cancer.

[12] Glenny AM, Gibson F, Auld E, Coulson S, Clarkson JE, Craig JV, Eden OB, Khalid T, Worthington HV, Pizer B (2010). The development of evidence-based guidelines on mouth care for children, teenagers and young adults treated for cancer. Eur J Cancer.

[13] Sung L, Robinson P, Treister N, Baggott T, Gibson P, Tissing W, Wiernikowski J, Brinklow J, Dupuis LL (2015). Guideline for the prevention of oral and oropharyngeal mucositis in children receiving treatment for cancer or undergoing haematopoietic stem cell transplantation. BMJ Support Palliat Care.

[14] Mahood DJ, Dose AM, Loprinzi CL, Veeder MH, Athmann LM, Therneau TM, Sorensen JM, Gainey DK, Mailliard JA, Gusa NL (1991). Inhibition of fluorouracil-induced stomatitis by oral cryotherapy. J Clin Oncol.

[15] Walladbegi J, Smith SA, Grayson AK, Murdoch C, Jontell M, Colley HE (2018). Cooling of the oral mucosa to prevent adverse effects of chemotherapeutic agents: an in vitro study. J Oral Pathol Med.

[16] Riley P, Glenny AM, Worthington HV, Littlewood A, Clarkson JE, McCabe MG (2015). Interventions for preventing oral mucositis in patients with cancer receiving treatment: oral cryotherapy. Cochrane Database System Rev.

[17] Peterson DE, Ohrn K, Bowen J, Fliedner M, Lees J, Loprinzi C, Mori T, Osaguona A, Weikel DS, Elad S, Lalla RV (2013). Systematic review of oral cryotherapy for management of oral mucositis caused by cancer therapy. Support Care Cancer.

[18] Svanberg A, Birgegard G, Ohrn K (2007). Oral cryotherapy reduces mucositis and opioid use after myeloablative therapy-a randomized controlled trial. Support Care Cancer.

[19] World Health Organization (1979) WHO handbook for reporting results of cancer treatment, vol 48. World Health Organization, Geneva, pp116–17

[20] Tomlinson D, Gibson F, Treister N, Baggott C, Judd P, Hendershot E, Maloney AM, Doyle J, Feldman B, Kwong K, Sung L (2010). Refinement of the Children’s International Mucositis Evaluation Scale (ChIMES): child and parent perspectives on understandability, content validity and acceptability. Eur J Oncol Nurs.

[21] Tomlinson D, Gibson F, Treister N, Baggott C, Judd P, Hendershot E, Maloney AM, Doyle J, Feldman B, Kwong K, Sung L (2009). Understandability, content validity, and overall acceptability of the Children’s International Mucositis Evaluation Scale (ChIMES): child and parent reporting. J Pediatr Hematol Oncol.

[22] Jacobs S, Baggott C, Agarwal R, Hesser T, Schechter T, Judd P, Tomlinson D, Beyene J, Sung L (2013). Validation of the Children’s International Mucositis Evaluation Scale (ChIMES) in paediatric cancer and SCT. Br Journal Cancer.

[23] Sprangers MA, Cull A, Groenvold M, Bjordal K, Blazeby J, Aaronson NK (1998). The European Organization for Research and Treatment of Cancer approach to developing questionnaire modules: an update and overview. EORTIC Quality of Life Study Group Qual Life Res.

[24] Hicks CL, von Baeyer CL, Spafford PA, van Korlaar I, Goodenough B (2001). The Faces Pain Scale-Revised: toward a common metric in pediatric pain measurement. Pain.

[25] McGrath PJ, Walco GA, Turk DC, Dworkin RH, Brown MT, Davidson K, Eccleston C, Finley GA, Goldschneider K, Haverkos L, Hertz SH, Ljungman G, Palermo T, Rappaport BA, Rhodes T, Schechter N, Scott J, Sethna N, Svensson OK, Stinson J, von Baeyer CL, Walker L, Weisman S, White RE, Zajicek A, Zeltzer L, PedIMMPACT (2008). Core outcome domains and measures for pediatric acute and chronic/recurrent pain clinical trials: PedIMMPACT recommendations. J Pain.

[26] von Baeyer CL, Spagrud LJ, McCormick JC, Choo E, Neville K, Connelly MA (2009). Three new datasets supporting use of the Numerical Rating Scale (NRS-11) for children’s self-reports of pain intensity. Pain.

[27] Todd KH, Funk JP (1996). The minimum clinically important difference in physician-assigned visual analog pain scores. Academ Emerg Med.

[28] Todd KH, Funk KG, Funk JP, Bonacci R (1996). Clinical significance of reported changes in pain severity. Ann Emerg Med.

[29] Tsze DS, Hirschfeld G, von Baeyer CL, Bulloch B, Dayan PS (2015). Clinically significant differences in acute pain measured on self-report pain scales in children. Acad Emerg Med.

[30] Heden LE, von Essen L, Ljungman G (2011). Effect of morphine in needle procedures in children with cancer. Eur J Pain.

[31] Schultz KF, Altman DG, Moher D (2010). CONSORT 2010 Statement: updated guidelines for reporting parallel group randomized trials. BMJ.

[32] DeSouza CM, Legedza AT, Sankoh AJ (2009). An overview of practical apporaches for handling missing data in clinical trials. J Biopharm Stat.

[33] Rubin DB (1987) Multiple imputation for nonresponse in surveys. Wiley, p 64

[34] Sorensen JB, Skovsgaard T, Bork E, Damstrup L, Ingeberg S (2008). Double-blind, placebo controlled, randomized study of chlorhexidine prophylaxis for 5-fluorouracil-based chemotherapy-induced oral mucositis with nonblinded randomized comparision to orall coolin (cryotherapy)in gastrointestinal malignancies. Cancer.

[35] Sato A, Saisho-Hattori T, Koizumi Y, Minegishi M, Iinuma K, Imaizumi M (2006). Prophylaxis of mucosal toxicity by oral propantheline and cryotherapy in children with malignancies undergoing myeloablative chemo-radiotherapy. Tohoku J Exp Med.

[36] Legert KG, Tsilingaridis G, Remberger M, Ringden O, Heimdahl A, Yucel-Lindberg T, Dahllof G (2015). The relationship between oral mucositis and levels of pro-inflammatory cytokines in serum and in gingival crevicular fluid in allogeneic stem cell recipients. Support Care Cancer.

[37] Legert KG, Remberger M, Ringden O, Heimdahl A, Dahllof G (2014). Reduced intensity conditioning and oral care measures prevent oral mucositis and reduces days of hospitalization in allogeneic stem cell transplantation recipients. Support Care Cancer.

[38] Jakobsen JC, Gluud C, Wetterslev J, Winkel P (2017). When and how should multiple imputation be used for handling missing data in randomised clinical trials - a practical guide with flowcharts. BMC Med Res Methodol.

