# Supplementary material for: Prevention of oral mucositis with cryotherapy in children undergoing hematopoietic stem cell transplantations—a feasibility study and randomized controlled trial
Source: Support Care Cancer. 2020 Jan 28;28(10):4869–79. doi: 10.1007/s00520-019-05258-2 (PMC7447624; doi:10.1007/s00520-019-05258-2)
Supplement: Supplementary file 1 — (PDF 79 kb) [file 520_2019_5258_MOESM1_ESM.pdf]

Online Recourse 1

Prevention of oral mucositis with cryotherapy in children undergoing hematopoietic stem cell transplantations-a randomized controlled trial.

Supportive Care in Cancer

Tove Kamsvåg, Annacarin Svanberg, Karin Garming-Legert, Johan Arvidson, Louise von Essen, Karin Mellgren, Jacek Toporski, Jacek Winiarski, Gustaf Ljungman.
Department of women's and children's Health, Pediatric onclogoly, Uppsala Univeristy, Sweden. Tove.kamsvag_magnusson@kbh.uu.se

**Question**		**Response alternatives**				
**1**	For how long during your chemotherapy infusion or the time instructed by the nurse did you cool your mouth?	All the time	More than half of the time	Less than half of the time	Not at all	
**1.1**	If not all the time, why did you not cool your mouth?	I forgot	It was unpleasant	I was sleeping	I didn't know I was supposed to	Other, please specify
**2**	Was it unpleasant for you to cool your mouth?	No, not at all	No, almost not at all	Yes, a little	Yes, very[a]	
**2.1**	In which way was it unpleasant to cool your mouth?	It was cold	It tasted bad	My mouth or teeth hurt	I got a headache	Other, please specify
**3**	Did the oral cryotherapy prevent you from doing other things during the therapy?	No, not at all	No, almost not at all	Yes, a little	Yes, very	
**3.1**	If yes, what were you not able to do?	Open question				

[a] Children who responded "*yes, very*" to question two were in the analysis considered to experience considerable discomfort of OC.

Online Recourse 1: *Oral cryotherapy evaluation questionnaire. The questionnaire was answered each day of chemotherapy/oral cryotherapy.*
