# Supplementary material for: Prevention of oral mucositis with cryotherapy in children undergoing hematopoietic stem cell transplantations—a feasibility study and randomized controlled trial
Source: Support Care Cancer. 2020 Jan 28;28(10):4869–79. doi: 10.1007/s00520-019-05258-2 (PMC7447624; doi:10.1007/s00520-019-05258-2)
Supplement: Supplementary file 2 — (PDF 63 kb) [file 520_2019_5258_MOESM2_ESM.pdf]

Online Recourse 2

Prevention of oral mucositis with cryotherapy in children undergoing hematopoietic stem cell transplantations-a randomized controlled trial.

Supportive Care in Cancer

Tove Kamsvåg, Annacarin Svanberg, Karin Garming-Legert, Johan Arvidson, Louise von Essen, Karin Mellgren, Jacek Toporski, Jacek Winiarski, Gustaf Ljungman.
Department of women's and children's Health, Pediatric onclogoly, Uppsala Univeristy, Sweden.
Tove.kamsvag_magnusson@kbh.uu.se

Conditioning regimen	Oral cryotherapy	Control	Total
TBI/Etoposide/Cy	9	5	14
TBI/Fludarabine/Cy	0	1	1
BEAC	0	1	1
BEAM	2	0	2
Busulfan/Cy	1	3	4
Busulfan/Fludarabine	6	3	9
Busulfan/Melphalan	4	2	6
Busulfan/Melphalan/Fludarabine	1	0	1
Fludarabine/Melphalan	0	1	1
Fludarabine/Cy	2	0	2
Treosulfan/Fludarabine/Thiotepa	1	3	4
Cy	2	1	5
Carmustine/Thiotepa/Etoposide	0	1	1
ATG	12	11	23

TBI-Total Body Irradiation, Cy-Cyclophosphamide, BEAM- Carmustine/Etoposide/ARA-C/Melphalan, BEAC-Carmustine/Etoposide/Ara-C/Cyclophosphamide, ARA-C-cytarabine, ATG-antithymocyte globuline

Online Recourse 2: *Conditioning regimens and use of Antithymocyt globulin for the children.*
